# Noncoding RNAs in Drug Resistance of Gastrointestinal Stromal Tumor

**DOI:** 10.3389/fcell.2022.808591

**Published:** 2022-01-31

**Authors:** Jiehan Li, Shuning Guo, Zhenqiang Sun, Yang Fu

**Affiliations:** ^1^ Department of Gastroenterology, The First Affiliated Hospital of Zhengzhou University, Zhengzhou, China; ^2^ Department of Colorectal Surgery, The First Affiliated Hospital of Zhengzhou University, Zhengzhou, China; ^3^ The Collaborative Innovation Center of Henan Province for Cancer Chemoprevention, Zhengzhou, China

**Keywords:** GIST, drug resistance, noncoding RNAs, targeted therapies, imatinib mesylate

## Abstract

Gastrointestinal stromal tumor (GIST) is the most common mesenchymal tumor in the gastrointestinal tracts and a model for the targeted therapy of solid tumors because of the oncogenic driver mutations in KIT and PDGDRA genes, which could be effectively inhibited by the very first targeted agent, imatinib mesylate. Most of the GIST patients could benefit a lot from the targeted treatment of this receptor tyrosine kinase inhibitor. However, more than 50% of the patients developed resistance within 2 years after imatinib administration, limiting the long-term effect of imatinib. Noncoding RNAs (ncRNAs), the non-protein coding transcripts of human, were demonstrated to play pivotal roles in the resistance of various chemotherapy drugs. In this review, we summarized the mechanisms of how ncRNAs functioning on the drug resistance in GIST. During the drug resistance of GIST, there were five regulating mechanisms where the functions of ncRNAs concentrated: oxidative phosphorylation, autophagy, apoptosis, drug target changes, and some signaling pathways. Also, these effects of ncRNAs in drug resistance were divided into two aspects. How ncRNAs regulate drug resistance in GIST was further summarized according to ncRNA types, different drugs and categories of resistance. Moreover, clinical applications of these ncRNAs in GIST chemotherapies concentrated on the prognostic biomarkers and novel therapeutic targets.

## Introduction

Gastrointestinal stromal tumors (GISTs), the most common mesenchymal tumor in the gastrointestinal (GI) tracts, are malignancies generated from the interstitial cells of Cajal (ICCs) or their undifferentiated precursors (Sircar et al., 1999; Min and Leabu, 2006). GIST, which occurs most commonly in the stomach and small intestine at middle and old age (Joensuu et al., 2001; Miettinen et al., 2002; von Mehren and Joensuu, 2018), has an incidence of 10 per million populations per year generally and comprises around 20% of the soft tissue sarcomas (Ducimetiere et al., 2011; Joensuu et al., 2013). Standard therapies for GIST confirmed by immunohistology are as the following: for the resectable GISTs without metastasis, surgical resection is the first choice; and for the unresectable, metastatic, or recurrent GISTs, administration of tyrosine kinase inhibitors (TKIs) is the primary approach (Nishida et al., 2008a; Li J. et al., 2017; Casali et al., 2022).

Gain-of-function mutations of the genes of KIT proto-oncogene receptor tyrosine kinase (KIT) or the platelet-derived growth factor receptor alpha (PDGFRA) resulted in the constitutive activation of these receptors, thus leading to the induction of GIST (Hirota et al., 1998; Rubin et al., 2001; Heinrich et al., 2003b) and imatinib mesylate (Gleevec), one receptor tyrosine kinase inhibitor, was found to competitively and effectively inhibit KIT and PDGFRA at submicromolar concentrations (Joensuu et al., 2002). Thus, imatinib mesylate is used and regarded as the first-line targeted therapy for GIST patients because of its durable responses in most tumors (Tuveson et al., 2001; Demetri et al., 2002). And the second-and third-line therapies for GIST patients consist of sunitinib malate (Sutent) and regorafenib (Stivarga), which are TKIs with activities against KIT, PDGFR and other targets like Raf-1 proto-oncogene serine/threonine kinase (RAF1), v-raf murine viral oncogene homolog B1 (BRAF) and fibroblast growth factor (FGFR) (Rock et al., 2007; Demetri et al., 2009; Demetri et al., 2013).

However, the resistance of these drugs widely exists in the therapy of patients with GISTs. There are about 14% of the patients with GISTs initially resistant to imatinib (Demetri et al., 2002). Additionally, within 2 years after the inception of the therapy based on imatinib in GIST, over 50% of the advanced and metastatic tumor patients developed secondary resistance (Gramza et al., 2009). When the resistance of imatinib occurred, second-line therapy including imatinib dose doubling and sunitinib administration was performed and third-line therapy of regorafenib was carried after the failure of second-line therapy (Serrano and George, 2020). And the novel TKIs, like sunitinib and regorafenib, showed a drug resistance trend, too (Heinrich et al., 2008a; Gajiwala et al., 2009; Garner et al., 2014; Ou B. et al., 2019). Therefore, it is of great significance to find the mechanisms involved in the drug resistance of GIST for further intervention to overcome the resistance.

Various genomic approaches have been used to understand the initiation, progression and drug resistance of multiple cancers (Hayes et al., 2014; Wang W.-T. et al., 2019; Yu et al., 2019). The genomic mutation landscapes have revealed that myriad mutations or copy number changes were frequently located in the noncoding regions of DNA when cancers were taken place (Guttman and Rinn, 2012). Functional noncoding RNAs (ncRNAs), like microRNAs (miRNAs), circular RNAs (circRNAs); small interfering RNAs (siRNAs), antisense RNAs (asRNAs), ribosomal RNAs (rRNAs), small nucleolar RNAs (snoRNAs), transfer RNAs (tRNAs), PIWI-interacting RNAs (piRNAs) and long noncoding RNAs (lncRNAs), are transcribed from noncoding DNAs which cover 95% of DNA sequences in the human genome (Esteller, 2011; Cech and Steitz, 2014). Furthermore, various studies showed that ncRNAs might play essential roles in anti-tumor drug resistances by changing drug efflux, regulating the tumor microenvironment and activating the signaling pathways (Bester et al., 2018; Wang W.-T. et al., 2019; Guo et al., 2020).

In GIST therapies, ncRNAs were also found to act in the process of drug resistance towards GISTs. For example, downregulation of lncRNA CCDC26 (Yan et al., 2019b) and miRNA-30a (Chen et al., 2020) contributes to imatinib resistance in GISTs. Thus, investigating how ncRNAs affect drug resistance might provide us with a new sight for GIST therapy. Here, we summarized the alterations of ncRNAs and different types of ncRNAs, drugs and resistance in the drug resistance process of GIST. We also discussed the functions and the potential clinical applications of ncRNAs during drug resistance in GIST.

## An Overview of Noncoding RNAs

The ncRNA family, which the majority of human genes are transcribed into, is a large and diverse class of RNAs with multiple functions (Dunham et al., 2012; Wong et al., 2018). These ncRNA molecules are simply classified relying on the cutoff of 200 nucleotides, which divides small ncRNAs from lncRNAs (Brandenburger et al., 2018). Amidst the different types of ncRNAs, miRNAs, circRNAs and lncRNAs are studied broadly nowadays.

MiRNAs, a group of single-stranded ncRNAs, are about 18–22 nt in length and act as post-transcriptional regulators of gene expression (Krol et al., 2010; Ameres and Zamore, 2013; Stepien et al., 2017). The mature miRNA strand is integrated into the RNA-induced silencing complex (RISC), and this protein complex has the ability to bind the complementary sequence in the 3′ untranslated regions (3′-UTR), which is dictated by the miRNA, and later silence its target genes by degrading the mRNA or blocking the mRNA translation (Sheu-Gruttadauria and MacRae, 2018). Additionally, a great many studies focused on miRNAs because of their crucial roles in the biological process (Shenouda and Alahari, 2009; Sun et al., 2018). Increasing evidence showed that miRNAs had functions in the cancer cell resistance towards chemotherapy and targeted therapies (Leonetti et al., 2019).

Unlike linear RNAs who have 5′ caps, 3′ tails and 5′ to 3′ polarity, circRNAs are circular, possessing covalently closed loop structures without either 5′ caps, 3′ tails or 5′ to 3′ polarity (Chen and Yang, 2015; Li et al., 2020). Functions of circRNAs in biological process are mainly mediated by regulating the alterative splicing (AS) and *cis*-transcription of RNAs, sequestering or scaffolding of macromolecules to act as competing endogenous RNAs (ceRNAs), and serving as translation templates (Lasda and Parker, 2014; Chen, 2020). Importantly, in the research of tumors, circRNAs have been found to involve and play essential roles in the proliferation, apoptosis, invasion and drug resistance (Zhang L. et al., 2019; Li et al., 2019). Moreover, circRNAs have unique functions and potential applications in the drug resistance through acting as a microRNA sponge and activating signaling pathways (Chen et al., 2019; Xu et al., 2020).

LncRNAs are longer than 200 nucleotides and comprise 80–90% of all mammalian noncoding transcriptome with low expression levels, poor interspecies conservation and high variance expression coefficient (Mercer et al., 2009; Yuan et al., 2020). Recent studies found that lncRNAs had the ability to regulate gene expression through various mechanisms like moderating the transcription of protein-coding genes, binding to proteins modulating their functions and conducting protein synthesis and RNA maturation (Wang and Chang, 2011; Carrieri et al., 2012). LncRNAs also participated widely in diverse physiological and pathological processes in human (Westra et al., 2018; Dallner et al., 2019). As important regulators of chromatin dynamics and gene regulation, lncRNAs correlated with countless cell signaling pathways and could influence multifarious factors including hormones, nutrients and age (Alvarez-Dominguez et al., 2014a; Alvarez-Dominguez et al., 2014b; Alvarez-Dominguez et al., 2015; Knoll et al., 2015). And with the studying of lncRNAs’ functions going on in recent years, mechanisms of lncRNAs on cancer drug resistance have been found a lot: they could affect the metabolizing enzymes, such as phase I and phase II enzymes, associate with oxidative stress and alter the epithelial-mesenchymal transition (EMT) in the process of drug resistance (Jiang et al., 2020; Liu et al., 2020). NcRNAs have played pivotal roles on drug resistance of multiple cancers which might be conducive to the research focusing on the chemotherapy and targeted therapy resistance.

## Molecular Characteristics of Gastrointestinal Stromal Tumors

**FIGURE 1:**
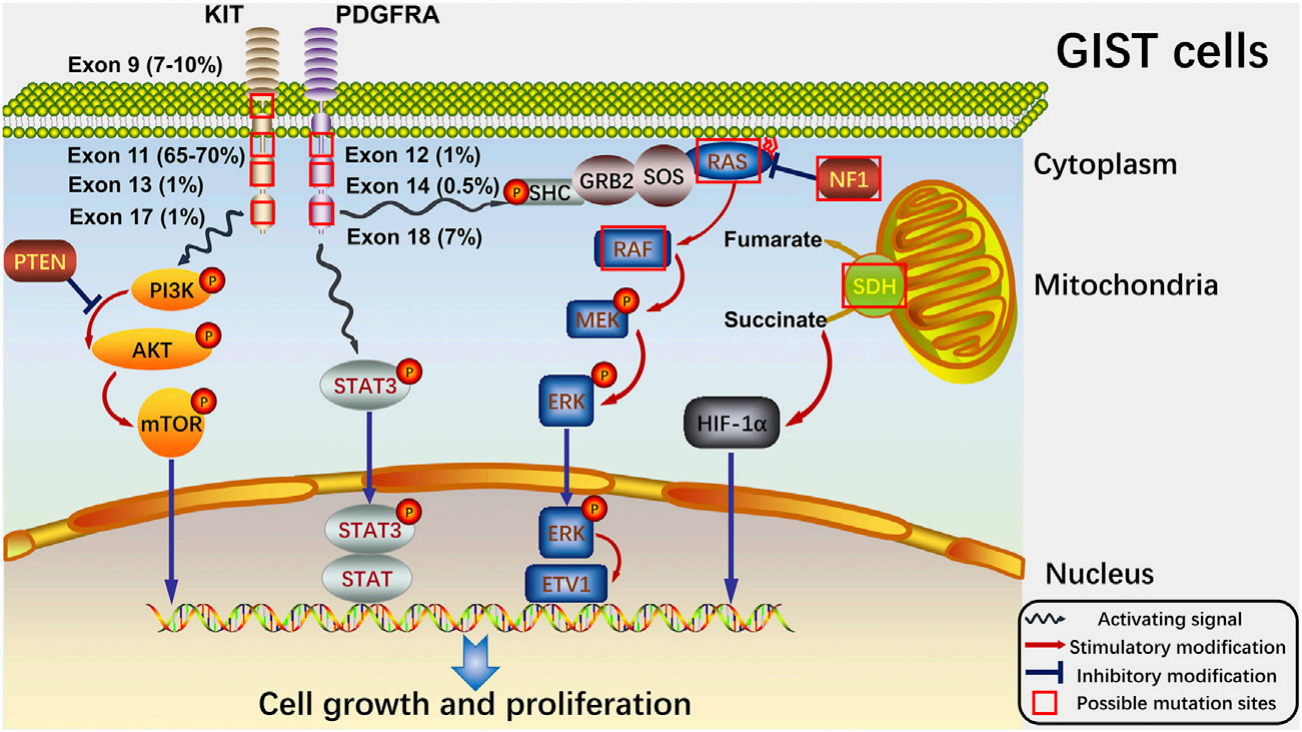
Signaling pathways of GISTs and potential sites where mutations always happen. At cellular level, the four main pathways (PTEN/PI3K/AKT/mTOR, STAT3, RAS/RAF/MEK/ERK and SDH/succinate/HIF-1α) and the significant proteins in the pathways are shown. And the possible mutation sites with their own mutation rates are also exhibited. The red box indicates the possible mutation sites. The blue wave-like arrow shows the activating signals from the receptor tyrosine kinase. And the red arrow shows the stimulatory modification between the proteins, while the blue “T” symbol shows the inhibitory modification.

Around 95% of GISTs stain positive for KIT in immunohistochemistry (Medeiros et al., 2004), and the sequencing data have shown that approximately 75% and 15% of the tumors harbored a gain-of-function mutation in KIT and PDGFRA, respectively. The activating mutations of KIT and PDGFRA are recognized as the major oncogenic drivers of GISTs (Hirota et al., 1998; Heinrich et al., 2003a; Corless et al., 2011; Wozniak et al., 2012). There are other gene mutations rarely occurring in GISTs, including mutations of set domain containing 2 (SETD2), succinate dehydrogenase (SDH), BRAF, RAS, neurofibromatosis type 1 (NF1), TP53, multiple endocrine neoplasia type 1 (MEN1) and retinoblastoma susceptibility gene (Rb1) (Huang et al., 2016; Merten et al., 2016; Shi et al., 2016a; von Mehren and Joensuu, 2018) (Figure 1).

KIT is a 145 kDa transmembrane glycoprotein from the receptor tyrosine kinase family and composes an extracellular domain, a transmembrane domain, a juxtamembrane domain and a tyrosine kinase domain encoded by exon 1-9, exon 10, exon 11 and exon 13–21, respectively (Lennartsson and Ronnstrand, 2012; Hemming et al., 2019). Stem cell factor (SCF), the ligand of KIT, induces the dimerization and activation of KIT, and then downstream signaling pathways of KIT, including phosphoinositide 3-kinase (PI3K) pathway, are stimulated to act in the process of differentiation, proliferation and survival (Zsebo et al., 1990; Hemesath et al., 1998; Timokhina et al., 1998; Palam et al., 2018). And mutations in KIT usually take place at exon 11 (90%) and exon 9 (8%) but seldom appear in exon 13 (1%) or exon 17 (1%) in GISTs (Joensuu et al., 2015; Joensuu et al., 2017). PDGFRA, whose mutations at exon 18 (D842V) and exon 12 respectively encode the activation loop and juxtamembrane domain of the tyrosine kinase (Cassier et al., 2012), is the other receptor tyrosine kinase and has a tight correlation with KIT at the molecular structure and chromosomal gene location (Heinrich et al., 2003b; Antonescu et al., 2005). Additionally, there are no significant differences between KIT and PDGFRA-mutant GISTs in the signaling pathways downstream, revealing that oncogenic signals presented by PDGFRA mutations in GIST are the same as KIT mutations (Heinrich et al., 2003a; Hirota et al., 2003; Corless et al., 2005). The remaining group of GISTs without KIT or PDGFRA mutations (5–10% of adult GISTs and 85% of pediatric GISTs) was termed “wild-type” in the past (Pappo and Janeway, 2009; Alkhuziem et al., 2017). And such GISTs have been recently found to harbor mutations concentrated on the NF1 gene or genes composed of SDH complex (Boikos et al., 2016; Ricci, 2016).

Besides, GISTs harboring mutations without KIT or PDGFRA would not be inhibited by the targeted therapy of imatinib (von Mehren and Joensuu, 2018). And when GIST contains the PDGFRA mutation at exon 18, imatinib or other permitted TKIs will not work, while Avapritinib (BLU-285), one type I inhibitor of KIT and PDGFRA activation loop mutants, could be effective at most times and is now undergoing regulatory assessment as the fourth-line treatment for GIST in United States (Cassier et al., 2012; Rose, 2017; Dhillon, 2020). Thus, targeted therapy of GIST depends on the clear judgment of molecular characteristics is of great significance for the use of TKIs. These molecular characteristics also help to analyze the drug resistance types in GISTs when the resistance takes place.

## Regulating Roles of Noncoding RNA in Drug Resistance of Gastrointestinal Stromal Tumors

**FIGURE 2:**
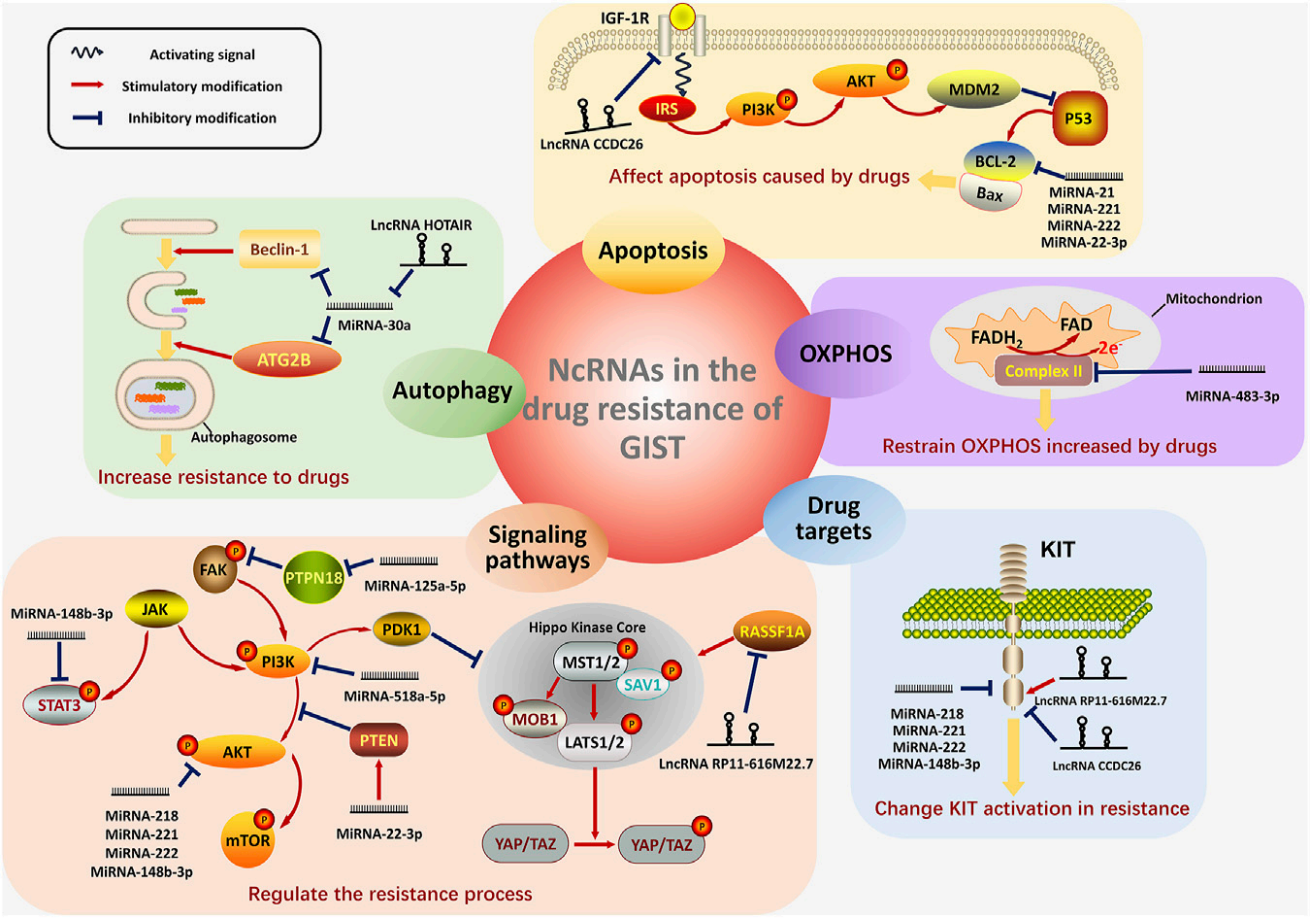
The four main mechanisms showing how ncRNAs work in the drug resistance of GIST. In GISTs, ncRNAs regulate the process of drug resistance mainly in the five mechanisms: OXPHOS (with purple background), autophagy (with green background), apoptosis (with orange background), drug target changes (with blue background) and activation of significant signaling pathways (with pink background). For the regulation of OXPHOS, the ncRNA regulate the Complex II on the inner membrane of mitochondrion. NcRNAs also play pivotal roles in the formation of autophagosome, thus modifying the resistance-induced autophagy. The signaling pathway of apoptosis are displayed and the ncRNAs involving in this process are shown, too. The figure in the lower right corner exhibits the transmembrane structure of KIT and the regulation of ncRNAs on KIT. Furthermore, the figures in the lower left corner show the main signaling pathways (PTEN/PI3K/AKT, JAK/STAT3, PTPN18/pFAK and RASSF1A/Hippo pathway), which are the targets of ncRNAs in the resistance. MiRNAs are distinguished from lncRNA by the different shapes. Activating signals from the receptor on the membrane are shown as the blue wave-like arrow. The red arrow represents the stimulatory modification between the proteins, and the blue “T” symbol shows the inhibitory modification.

In GISTs, NcRNAs play pivotal regulating roles in drug resistance through oxidative phosphorylation, autophagy, apoptosis, changing drug targets and activating signaling pathways (Figure 2).

### NoncodingRNAs Regulate Oxidative Phosphorylation in Drug Resistance of Gastrointestinal Stromal Tumors

Deregulated cell metabolism was one of the predominant features of cancer (Hanahan and Weinberg, 2011; Zanotelli et al., 2021). Although the metabolic reprogramming of cancer is mainly characterized as Warburg effect, increasing evidence recently revealed that enhanced dependence on oxidative phosphorylation (OXPHOS) of cancer cells appeared during cancer progression (Viale et al., 2014; Faubert et al., 2020). Furthermore, metabolic adaption from glycolysis to OXPHOS played pivotal roles in the drug resistance of cancer cells. In acute myelogenous leukemia, cancer cells which were treated with cytarabine or initially cytarabine resistant possessed enhanced OXPHOS and OXPHOS inhibition showed raised cytarabine sensitivity (Farge et al., 2017). And in chronic lymphocytic leukemia, after the appearance of acquired resistance of venetoclax, the inhibitor of B-cell lymphoma-2 (BCL-2), mitochondrial mass of cancer cells and OXPHOS increased and OXPHOS inhibition rescued the venetoclax sensitivity (Guieze et al., 2019).

Studies of Vitiello et al. (Vitiello et al., 2018) and Huang et al. (Huang et al., 2020) found that the OXPHOS levels of mitochondrial respiratory complexes, like Complex II, III, and V, were upregulated in GIST cells after imatinib treatment and inhibiting OXPHOS could increase the imatinib sensitivity in GISTs, which showed the potential functions of metabolic reprogramming towards imatinib treatment. Moreover, the roles of microRNAs in regulating OXPHOS towards imatinib resistance of GISTs were recently explored. Huang et al. found that miRNA-483-3p, whose expression was downregulated in imatinib-treated GIST samples, could inhibit the protein expression of mitochondrial respiratory Complex II, thus increasing the cell death induced by imatinib in GIST cells (Huang et al., 2021). OXPHOS played a pivotal role in the drug resistance of GIST and the detailed mechanisms between cell metabolism and ncRNAs in the drug resistance need further investigation.

### NoncodingRNAs Moderate Autophagy in Drug Resistance of Gastrointestinal Stromal Tumorss

Autophagy, whose major acting procedures include activation, vesicle nucleation and elongation, fusion and degradation, is a self-degradative system and plays pivotal roles in the metabolism of cells and the body (Kim and Lee, 2014). Enormous structures in cells would be degraded by autophagy, which benefits the cells’ survival via recycling their composing metabolites (Smith and Macleod, 2019). Autophagy widely exists in the progression of tumor. It supports nutrient reuse and metabolic balance, thus affecting the tumor genesis and development (Bu et al., 2021). And for the tumor chemotherapy, studies showed that constructive autophagy prevented cell death from tumor chemotherapy and brought about drug resistance and recurrence of tumors (Buchser et al., 2012; Li Y.-J. et al., 2017). Moreover, studies have demonstrated that autophagy was a stress response to avoid the imatinib-induced starvation in GISTs and chronic myeloid leukemia (Gupta et al., 2010; Ravegnini et al., 2017; Mitchell et al., 2018; Han et al., 2019; Zheng et al., 2020; Zhang et al., 2021). And autophagy inhibition by lysosomotropic agents could reduce the imatinib resistance of GISTs *in vitro* and *in vivo* (Rubin and Debnath, 2010; Burger et al., 2015). It could be concluded from these studies that autophagy plays protective roles in the imatinib-induced cell death of GISTs, while the inhibition of autophagy could overcome the resistance, to a certain extent.

Accumulating evidence illustrated that ncRNAs, including miRNAs, lncRNAs and circRNAs, could engage in not only the transcriptional but the post-transcriptional regulation of genes relevant to autophagy through the regulatory networks of autophagy (Frankel et al., 2017; Yao et al., 2019). Different expression levels of ncRNAs determine the levels of autophagy at various physiological and pathological stages, including cellular senescence, cancer genesis and drug resistance (Zhang et al., 2017; Wang J. et al., 2019; Bermúdez et al., 2019; Xu et al., 2019).

In the imatinib resistance of GISTs, a research concentrating on the functions of miRNA-30a has found that autophagy caused by imatinib exposure in GIST cell lines was correlated with miRNA-30a (Chen et al., 2020). Beclin-1, the mammalian autophagy gene, which has also been proved as a tumor suppressor, was found to be directly targeted by MiRNA-30a and served as a link between miRNA-30a and autophagy in GISTs (Liang et al., 1999; Chen et al., 2020). Moreover, miRNA-30a increased the sensitivity of imatinib in GIST cells by down-regulating the expression of Beclin-1. Additionally, studies of Zhang et al. reported that lncRNA-HOTAIR activated autophagy by the miRNA-130a/autophagy-related protein 2 homolog B (ATG2B) pathway, thus promoting the imatinib resistance of GIST cells (Zhang et al., 2021). Generally speaking, ncRNAs could adjust the process of drug resistance in GISTs by regulating the expression levels of proteins or pathways related to autophagy.

### NoncodingRNAs Adjust Apoptosis in Drug Resistance of Gastrointestinal Stromal Tumorss

Diverse studies revealed that cell death through apoptosis pathways could be generated by the larger part of cancer chemotherapy drugs. And once the apoptosis is disorganized, drug resistance and increased cancer cell survival appear (Mohammad et al., 2015; Das et al., 2018; Mou et al., 2018). In the drug resistance of GISTs, there are a great many studies exhibiting that functions of ncRNAs on the resistance are associated with apoptosis.

LncRNA CCDC26 knocking down decreased the apoptosis of GIST cells treated with imatinib through upregulating insulin-like growth factor 1 receptor (IGF-1R), which acted in the apoptosis pathways (Li et al., 2018; Yan et al., 2019b; Zhang Y. et al., 2019). Additionally, lncRNA CCDC26 was also reported to interact with c-KIT, thus affecting the rates of apoptotic cells caused by imatinib (Cao K. et al., 2018). BCL-2, whose gene families play pivotal roles in the programmed cell death regulations, induces apoptosis evasion and drug resistance evolution in cancers (Ashkenazi et al., 2017; Maji et al., 2018). In GISTs, BCL-2 was down-regulated by miRNA-21, miRNA-221 and miRNA-222, whose mimics were transfected in GIST cells thus significantly aggravating the apoptosis motivated by imatinib (Ihle et al., 2015; Cao et al., 2016). The overexpression of miRNA-518a-5p increased the proportion of apoptotic GIST cells treated with imatinib, which advocated that miRNA-518a-5p acted a part in interfering with the imatinib resistance (Shi Y. et al., 2016). When treated with imatinib, the resistance generated by inhibiting the apoptosis of GISTs could be attenuated by multiple ncRNAs through targeting different apoptosis-related proteins.

### NoncodingRNAs Affect Drug Targets in Drug Resistance of Gastrointestinal Stromal Tumorss

Drug resistance in GISTs mainly concentrated on the primary and secondary resistance of imatinib (Joensuu et al., 2013). And the primary resistance is correlated with several genotypes of GISTs, such as mutations of KIT exon 9 whose receptor dimerization without ligand hinders the binding of imatinib structurally (Heinrich et al., 2003a) and mutations of PDGFRA D842V located in exon 18 (Lasota et al., 2004). The secondary resistance mainly happens via the acquirements of secondary mutations on KIT under the imatinib pressure and leads to the pathological activation of downstream signaling pathways like the PI3K/protein kinase B (PKB, AKT) pathway (Liegl et al., 2008; Pierotti et al., 2011).

Recent research revealed that ncRNAs in GISTs could alter the resistance of imatinib by directly targeting and reducing the activation of KIT. At protein expression levels, exogenous miRNA-221 and miRNA-222 reduced phosphorylated KIT and total KIT levels in GISTs (Ihle et al., 2015). And phosphorothioation and 2′-O-methylation of miRNA-221 and miRNA-222 in GIST cells were reported to effectively inhibit KIT expression, thus affecting various cellular processes mediated by KIT, including resistance of imatinib (Durso et al., 2016). Another research found the direct regulation relationship between miRNA-222 and KIT in imatinib-resistant GIST cells whose proliferation ability was inhibited by miRNA-222 (Gits et al., 2013). In GISTs, KIT was also targeted directly by miRNA-218 and miRNA-148b-3p, which acted as tumor suppressors and sensitized GIST cells to imatinib therapy (Fan et al., 2014; Wang Y. et al., 2018). Moreover, through collaborating with c-KIT, knocking down lncRNA CCDC26 and overexpressing lncRNA RP11-616M22.7 increased the resistance of GIST cells to imatinib (Cao K. et al., 2018; Shao et al., 2021). The microarrays which screened tumour-specific circRNA profiles of GIST, identified a GIST-specific circRNA-miRNA-mRNA regulatory network related to KIT, which showed the potentials of circRNAs in the drug resistance of GIST by targeting KIT (Jia et al., 2019). By directly affecting the drug targets in GISTs, ncRNAs play essential roles in drug resistance.

### NoncodingRNAs Activate Signaling Pathways in Drug Resistance of Gastrointestinal Stromal Tumorss

As a critical signaling pathway adjusting various biological processes, phosphatase and tensin homolog (PTEN)/PI3K/AKT signaling conducts cell proliferation, apoptosis and invasion in GI tract tumors, especially in GISTs (Carnero et al., 2008; Long et al., 2018; Hu et al., 2019). Abnormal expression of PTEN/PI3K/AKT signaling was exhibited as being conducive to the drug resistance of targeted therapies caused by stimulating mutations of PI3K-related genes, including PIK3C2A, to improve the cell proliferation regulated by the growth factors, invasion and metastasis (Hafsi et al., 2012). PTEN/PI3K/AKT pathway was also found to regulate and participate in the drug resistance process in GISTs. Serving as the gene-specific target of PIK3C2A, miRNA-518a-5p downregulated PIK3C2A in GISTs to alter the cellular response towards imatinib, thus lessening the resistance (Shi Y. et al., 2016). And miRNA-218, miRNA-148b-3p, miRNA-221 and miRNA-222 were found to increase the sensitivity of GISTs towards imatinib through targeting PI3K/AKT pathway (Fan et al., 2015; Ihle et al., 2015; Wang Y. et al., 2018). Moreover, in the resistance process of cisplatin in GISTs, PTEN/PI3K/AKT pathway also played pivotal roles. MiRNA-22-3p could increase the chemosensitivity of cisplatin in GIST cell lines by targeting the PTEN/PI3K/AKT pathway (Xu et al., 2018).

Also, the integrin-mediated signaling transduction pathway of focal adhesion kinase (FAK) was identified by the Gene Expression Omnibus (GEO) database and Kyoto Encyclopedia of Genes and Genomes (KEGG) pathway enrichment analysis as the pathway where the significantly different ncRNAs between the imatinib treated GIST patients with and without resistance were enrich in (Zhang et al., 2018). Moreover, FAK is verified as the downstream pathway of PTPN18 contributing to the imatinib resistance in GISTs, which is regulated by miRNA-125a-5p (Akcakaya et al., 2014; Huang et al., 2018). MiRNA-125a-5p decreased the expression levels of PTPN18, thus increasing the phosphorylation of FAK in the imatinib-resistant GIST cell lines (Huang et al., 2018).

The Janus kinase (JAK)-signal transducer and activator of transcription 3 (STAT3) signaling pathway, which was activated abnormally in various cancers to increase the proliferation, survival, drug resistance and metastasis of cancer cells (Wang T. et al., 2018; Johnson et al., 2018), was identified from the KEGG pathway analysis, based on ncRNAs with significantly different expression levels selected by the genome-scale clustered regularly interspaced short palindromic repeats (CRISPR)-Cas9 knockout screening in GISTs with imatinib resistance (Cao J. et al., 2018). STAT3 was also found to be downregulated by miRNA-148b-3p in GISTs, thus reducing the imatinib resistance of GIST cells (Wang Y. et al., 2018). Gene set enrichment analysis (GSEA) also identified JAK-STAT3 signaling pathway as one of the interfered signaling pathways of lncRNA RP11-616M22.7, which was increased in the imatinib-resistant GIST samples (Shao et al., 2021).

As a critical signaling pathway involving in a broad range of cancers, Hippo pathway controls multiple cellular functions and tissue-level processes crucial to the cancer progression and therapy, like cell proliferation, cell survival, drug resistance, stem cell phenotype, planar and apicobasal cell polarity, cell–cell adhesion, contact inhibition, metastasis and so on (Harvey et al., 2013). And in GISTs, deregulation of the Hippo pathway also occurs (Ou W.-B. et al., 2019; Guerin et al., 2020). Hippo pathway acted as an oncogenic regulator in KIT-independent GISTs by inducing the expression of Cyclin D1, which was the oncogenic mediator for primary imatinib resistant and untreatable GISTs (Ou W.-B. et al., 2019; Chen et al., 2021). NcRNAs targeting the Hippo pathway also involved in the malignant transformation of GISTs (Yin et al., 2021). In the drug resistance of GISTs, lncRNA RP11-616M22.7 promoted GIST resistance to imatinib by interacting with the Ras association domain family protein1 isoform A (RASSF1A), which is an important upstream regulator of the Hippo pathway (Shao et al., 2021). And through specifically binding to RASSF1A thus inhibiting its function, lncRNA RP11-616M22.7 decreased the expression of mammalian STE20-like kinase (MST)-1, MST2, large tumor suppressor kinase (LATS)-1, and LATS2, and increased the expression of Yes-associated protein (YAP) and transcriptional coactivator with PDZ-binding motif (TAZ), thus promoting the cell proliferation and inhibiting the apoptosis under imatinib treatment (Shao et al., 2021). More key signaling pathways relevant to ncRNAs would be found in future studies on drug resistance of GISTs.

## Different Types of NoncodingRNAs on the Drug Resistance of Gastrointestinal Stromal Tumorss

### MicroRNAs

Among the different kinds of ncRNAs, miRNAs are studied most broadly in the resistance of targeted therapies (Corra et al., 2018; Wang W.-T. et al., 2019). In GISTs, there are also a great deal of miRNAs relating to drug resistance. Recently, miRNA-221 and miRNA-222, whose chemical modifications affected the proliferation of imatinib-resistant GIST cells (Durso et al., 2016), were found to target KIT and promoted apoptosis of GIST cells through the KIT/AKT pathway during the imatinib resistance (Gits et al., 2013; Ihle et al., 2015). Another miRNA, miRNA-125a-5p, was also an important ncRNA in the drug resistance of GISTs. Studies by Lui et al. revealed that overexpression of miRNA-125a-5p suppressed the expression of PTPN18 and increased the phosphorylation of FAK, thus contributing to the re-sensitization of GIST cells towards imatinib (Akcakaya et al., 2014; Huang et al., 2018). One research based on the GEO database (GSE63159 and GSE45901) also identified miRNA-125a-5p as a critical ncRNA in patients with GISTs treated by the targeted therapy of imatinib (Zhang et al., 2018). Moreover, reducing influence of miRNA-218 on imatinib resistance was proved by Fan et al. (Fan et al., 2014; 2015). There were also other miRNAs, such as miRNA-483-3p (Huang et al., 2021), miRNA-518e-5p (Kou et al., 2018), miRNA-518a-5p (Shi Y. et al., 2016), miRNA-148b-3p (Wang Y. et al., 2018), miRNA-17-92 (Gits et al., 2013), miRNA-21 (Cao et al., 2016), miRNA-23b (Cao J. et al., 2018), miRNA-30a (Zheng et al., 2015), miRNA-320a (Gao et al., 2014), miRNA-505 (Cao J. et al., 2018), miRNA-92a-3p (Amirnasr et al., 2019) and miRNA-99a-5p (Amirnasr et al., 2019), being identified or demonstrated that they played important roles in the process of imatinib resistance in GISTs.

### Long NoncodingRNAs

LncRNA, another kind of ncRNA, has been considered as a new mechanism of drug resistance nowadays and attracted full attention for researching various cancers. Through upregulating IGF-1R and c-KIT to affect the cell viability, proliferation, and apoptosis of imatinib treated GIST cells, downregulation of lncRNA CCDC26 promoted the process of imatinib resistance which could be used to develop potential strategies overcoming the imatinib resistance of GIST patients (Cao K. et al., 2018; Yan et al., 2019b). Multidrug resistance protein (MRP)-1 is an ATP-binding cassette transporter that participated in the resistance of many drugs like anti-cancer drugs, opiates and antibiotics (Johnson and Chen, 2017) and also played essential roles in the chemotherapy of GISTs (Plaat et al., 2000; Theou et al., 2005). The lncRNA HOTAIR, which considerably reduced the MRP1 expression, was found to have the possibility of inactivating the PI3K/AKT pathway, thus decreasing the resistance of imatinib treatment (Dai et al., 2017; Wang et al., 2017). However, the lncRNA HOTAIR whose distribution was transferred from nucleus to cytoplasm after imatinib treatments was reported to increase imatinib resistance through activating autophagy by the miRNA-130a/ATG2B pathway in GISTs by Zhang et al. (Zhang et al., 2021). The exact function of lncRNA HOTAIR needs to be further explored in the drug resistance in GIST. Additionally, one study based on the microarray identified lncRNA-DNAJC6-2 together with the hypoxia-inducible factor-1 (HIF-1) pathway as drug targets. And lncRNA-DNAJC6-2 probably played a part in the secondary imatinib-resistant GISTs by targeting the translational factors, including TBP, TAF1, NRF1, MAX, STAT3 and E2F6, which regulate the translation process of mRNA (Yan et al., 2019a). LncRNA RP11-616M22.7 was also found to regulate the imatinib resistance of GISTs through the Hippo pathway (Shao et al., 2021). These researches showed that lncRNA, as a noncoding RNA, is closely related to drug resistance in GISTs, which might be applied as novel therapeutic strategies against GISTs.

## Dual Regulations of NoncodingRNAs on Drug Resistance in Gastrointestinal Stromal Tumorss

**FIGURE 3:**
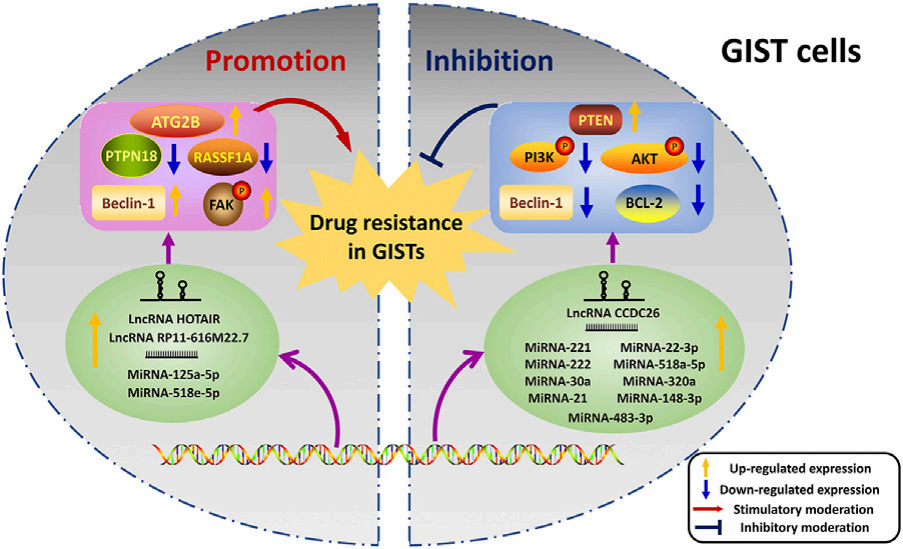
Dual regulations of ncRNAs on drug resistance in GISTs. The left half shows the promoted effects of ncRNAs on drug resistance, and the inhibited effects of ncRNAs are exhibited on the right half. NcRNAs transcribed from DNA regulate their targets thus playing roles in the resistance. The yellow arrow indicates that the expression levels of proteins or ncRNAs are up-regulated. And the blue arrow reveals the down-regulated expression levels of proteins or ncRNAs. The red arrow represents the stimulatory moderations, and the blue “T” symbol shows the inhibitory moderations.

The functions of ncRNAs mainly concentrated on the promotion and inhibition aspects during the regulation of drug resistance in GISTs (Figure 3).

### Promotion of NoncodingRNAs on Drug Resistance

As positive regulators of drug resistance, various ncRNAs were proved to promote drug resistance in numerous cancer types (Loewen et al., 2014; Qu et al., 2016; Ren et al., 2018). And in GISTs, one study of miRNA-125a-5p focused on its promoting effects in the imatinib resistance (Akcakaya et al., 2014). In this study, the overexpression of miRNA-125a-5p silenced the expression of PTPN18, whose expression level was negatively correlated with cell viability in GIST cells under the exposure of imatinib. Additionally, miRNA-125a-5p was demonstrated to correlate with metastasis and mutational status of KIT in GISTs by Kaplan Meier survival and log-rank analyses. Moreover, the promoted effects of miRNA-125a-5p and its downstream regulations were ulteriorly studied (Huang et al., 2018). Expression of phosphorylated FAK (pFAK), which was higher in the imatinib-resistant GIST subclones, could be incresed by miRNA-125a-5p and PTPN18 silencing in GIST cells. Suppression of pFAK by FAK inhibitor 14 was shown to rescue the imatinib resistance caused by miRNA-125a-5p overexpression. Up-regulation of miRNA-125a-5p and pFAK promoted drug resistance in GISTs. Moreover, expression levels of miRNA-518e-5p in the serum were positively correlated with imatinib resistance in GISTs (Kou et al., 2018). The lncRNA HOTAIR and RP11-616M22.7 were also proved to increase the resistance of imatinib in GISTs (Shao et al., 2021; Zhang et al., 2021). NcRNAs might promote drug resistance which could be applied as therapy targets against drug resistance in GISTs.

### Inhibition of NoncodingRNAs on Drug Resistance

NcRNAs are also served as negative regulators in the resistance of chemotherapies (Fanini and Fabbri, 2016; Jin et al., 2019). Inhibited functions of ncRNAs on drug resistance are universal in GISTs. It is reported that GIST cells which possessed high expression levels of lncRNA CCDC26 were more sensitive to imatinib than those GIST cells with the lower levels, and knockdown of lncRNA CCDC26 obviously increased imatinib resistance. Further studies showed that the downregulation of lncRNA CCDC26 helped to the imatinib resistance in GISTs by upregulating IGF-1R and interacting with c-KIT (Cao K. et al., 2018; Yan et al., 2019b). MiRNA-221 and miRNA-222 targeted KIT and restrained the KIT/AKT signaling pathway to inhibit cell proliferation, modify the progression of the cell cycle, induce apoptosis and reduce resistance in imatinib-resistant GIST cells (Gits et al., 2013; Ihle et al., 2015). In addition, imatinib treatment was found to reduce the expression levels of miRNA-30a, which was found to inhibit the imatinib resistance of GIST cells by targeting Beclin-1, thus lessening the imatinib resistance related autophagy (Chen et al., 2020). Moreover, ncRNAs like miRNA-483-3p, miRNA-21, miRNA-22-3p, miRNA-518a-5p, miRNA-320a, miRNA-218, and miRNA-148b-3p acted as negative regulators to enhance the drug sensitivity and played significant roles in the process of GIST drug resistance (Gao et al., 2014; Shi Y. et al., 2016; Cao et al., 2016; Wang Y. et al., 2018; Xu et al., 2018; Huang et al., 2021). These drug resistance-inhibiting ncRNAs could be served as small molecular drugs against drug resistance in GISTs in the future.

## Effect of NoncodingRNAs on Resistance of Various Drugs for Gastrointestinal Stromal Tumorss

### Resistance of Imatinib

Imatinib mesylate has become the first-line agent for GISTs from its approval in 2002 as a TKI against KIT, PDGFRA, Abelson tyrosine kinase (ABL), FMS-like tyrosine kinase-3 (FLT-3) and colony stimulating factor-1 receptor (CSF1R) (Demetri et al., 2002; Bauer and Joensuu, 2015). The usage of imatinib improved the outcome of patients with untreatable advanced and metastatic GIST extensively. Nevertheless, primary resistance occurs in 14% of the patients, and over 50% of the remaining patients would develop secondary resistance within 2 years (Demetri et al., 2002; Gramza et al., 2009). A great deal of studies reported the influence of ncRNAs in the process of imatinib resistance.

In the imatinib-resistant cell line GIST48, miRNA-221 mimic transfection markedly reduced cell proliferation and induced apoptosis related to imatinib (Gits et al., 2013; Ihle et al., 2015). Interestingly, modified miRNA-221 was also found to inhibit the expression of the KIT gene effectively, which might be used in the solution to overcome imatinib resistance in GIST (Durso et al., 2016). Additionally, high expression of miRNA-148b-3p suppressed cell proliferation synergistically with the effect of imatinib, causing sensitivity to imatinib treatment in GIST cells (Wang Y. et al., 2018). In parallel, Chen et al. demonstrated that miRNA-30a could decrease the autophagy level to sensitize GIST cells to imatinib mesylate by targeting Beclin-1 (Chen et al., 2020). A study by Cao et al. showed the potential role of miRNA-23b and miRNA-505 during imatinib resistance by performing the genome-scale CRISPR-Cas9 knockout screening in the GIST cell lines with and without imatinib resistance (Cao J. et al., 2018). It can be concluded that ncRNAs played essential roles in the imatinib resistance of GISTs, and the clinical applications need to be further explored.

### Resistance of Cisplatin

Cisplatin, the best-known platinum-based drug, is used in the chemotherapy of multiple solid tumors, including reproductive system cancers, head and neck cancers, lung cancers and cancers in the GI tracts (Prestayko et al., 1979; Galluzzi et al., 2012). The most widely studied mechanism of how cisplatin acting on cancers is activating the DNA damage response of cancer cells (Galluzzi et al., 2012). Treated with cisplatin, DNAs in cancer cells form adducts to inhibit transcription and synthesis. This process sets off a cascade of intracellular signaling transduction to eliminate the disease (Siddik, 2003). However, cisplatin resistance, including intrinsic and acquired resistance, introduces itself rapidly, thus causing therapeutic failure and recurrence. Cancer cells resist the cell death caused by cisplatin by reducing the uptake of cisplatin, strengthening the efflux of cisplatin and invalidating cisplatin through covalent binding with glutathione or metalloproteins (Zisowsky et al., 2007; Galluzzi et al., 2012; Rocha et al., 2014).

In GIST cells, the chemosensitivity of cisplatin was found to be increased by miRNA-22-3p through activating PTEN/PI3K/AKT pathway (Xu et al., 2018). The research of Xu et al. pointed out that GIST cells administrated with both miRNA-22-3p mimics and cisplatin would have lower survival rate and BCL-2/BCL-2 associated X (Bax) ratio, and higher apoptosis rate and Caspase-3 level than those treated with cisplatin merely (Xu et al., 2018). Though cisplatin was not used as the first-line agent for GIST therapy in the clinic, analyzing and probing into the functions of ncRNAs on the cisplatin resistance in GIST provided us with more information about the chemotherapy of GIST and new strategies to overcome the drug resistance dilemma in GIST.

## Effect of NoncodingRNAs on Different Types of Drug Resistance in Gastrointestinal Stromal Tumorss

Primary resistance symbolizes the initial resistance of the drugs when the drugs are first applied for the treatment, while the secondary resistance represents that the resistance gradually takes place after a period of successful drug treatment.

### Primary Resistance

There are 10-15% of GIST patients initially failing to react to the imatinib exposure, and the early progression always occurs in 3–6 months after the initial exposure (Sleijfer et al., 2007; Blanke et al., 2008). It was widely considered that patients with primary resistance to imatinib harbored drug target mutations such as KIT mutation in exon 9, wild-type KIT mutations and PDGFRA point mutation D842V (Heinrich et al., 2003a; Heinrich et al., 2008b). To overcome the primary resistance, one should target KIT, which is the target of imatinib, but in a different way rather than imatinib does.

And in GISTs, ncRNAs, whose functions concentrated on the KIT, played essential roles in the primary resistance. It was reported that miRNA-17-92, miRNA-221, miRNA-222 and miRNA-148b-3p induced apoptosis and cell cycle regulation of primary imatinib-resistant GIST cells by directly targeting KIT (Gits et al., 2013; Ihle et al., 2015; Wang Y. et al., 2018). One study based on the microRNA profiles of 53 GIST samples was conducted to identify the differentially expressed miRNAs among the imatinib-sensitive and primary imatinib-resistant GISTs, and miRNAs-mRNAs interaction networks were drawn to show the biochemical pathways and gene regulations in the process of resistance (Amirnasr et al., 2019). LncRNA CCDC26 also played pivotal roles in the primary resistance of imatinib by regulating IGF-1R in GISTs, which could help the targeted treatment (Yan et al., 2019b). In other researches which focused on primary drug resistance in GISTs, lncRNA RP11-616M22.7, miRNA-22-3p, miRNA-218, and microRNA-30a showed potential functions, including changing drug targets, activating relevant pathways and inactivating autophagy in the primary resistance (Fan et al., 2015; Xu et al., 2018; Chen et al., 2020; Shao et al., 2021).

### Secondary Resistance

Secondary resistance of imatinib happens more frequently than the primary resistance in GIST, with over 50% of the imatinib administrated GIST patients developing secondary resistance within 2 years after the beginning of the imatinib therapy (Demetri et al., 2002; Gramza et al., 2009; Wang et al., 2011). A second mutation in KIT, the same allele as primary resistance happening, is the most universal reason for the secondary resistance, and such a mutation has not been observed in the tumors with primary resistance yet (Chen et al., 2004; Antonescu et al., 2005). Secondary mutations usually appeared at exons 13 and 17 of KIT, and these mutations changed the tyrosine kinase domains to inhibit the binding of imatinib and made the catalytic site related to imatinib insensitive (Chen et al., 2004; Nishida et al., 2008b). In addition, multiple secondary mutations have been found to appear on KIT along with the tumor progression, even in the same tumor nodule. The intra- and inter-lesional heterogeneity of the secondary mutations in GIST are extensive, which lead to the therapeutic difficulties of imatinib. And this is where the selection of other more target-specific TKIs comes in (Heinrich et al., 2006; Wardelmann et al., 2006; Liegl et al., 2008).

Nowadays, functions of ncRNAs in the secondary resistance on imatinib in GISTs have attracted full attention. A series of studies based on the microarray and qRT-PCR analysis identified the differential expression levels of miRNAs between imatinib treated GIST patients with and without secondary resistance and highlighted the functional role of miRNA-125a-5p on secondary imatinib resistance by regulating PTPN18 to affect the phosphorylation of FAK (Akcakaya et al., 2014; Huang et al., 2018). MiRNA-483-3p inhibited the mitochondrial respiratory complexes to restrain the OXPHOS caused by imatinib resistance in GIST cells (Huang et al., 2021). MiRNA-518 family members miRNA-518a-5p and miRNA-518e-5p were also proved to play an important part in the secondary imatinib resistance through targeting PIK3C2A and affecting the cellular response to imatinib in GISTs (Shi Y. et al., 2016; Kou et al., 2018). Moreover, lncRNA CCDC26, whose expression levels time-dependently reduced by the imatinib exposure, sensitized the secondary resistant GIST cells to imatinib by inhibiting c-KIT (Cao K. et al., 2018). Additionally, significant ncRNAs in the secondary resistance in GISTs could also be identified by techniques like genome-scale CRISPR-Cas9 knockout screening (Cao J. et al., 2018), databases like the GEO database (Zhang et al., 2018) and gene functions analyses like KEGG pathway analysis and Gene Ontology enrichment analysis (Yan et al., 2019a). NcRNAs have definite functions in the secondary resistance of GIST, which provides us with a novel insight into the targeted therapies for GISTs.

## Clinical Applications and Possible Research Directions of NoncodingRNAs in Gastrointestinal Stromal Tumorss

### Act as Potential Prognostic Biomarkers

Aside from the functions and mechanisms of ncRNAs in the chemotherapy resistance as discussed above, prognostic roles of ncRNAs in GISTs are also shown. Numerous ncRNAs have been revealed as prognostic biomarkers in multiple kinds of tumors such as gastric cancer (Wei et al., 2020), non-small cell lung cancer (NSCLC) (Leonetti et al., 2019), hepatocellular carcinoma (Wei et al., 2019), myeloma (Rastgoo et al., 2017) and leukemia (Bhat et al., 2020). And in GISTs, ncRNAs might be considered as a biomarker for recurrence, metastasis and drug resistance as well. Kou et al. analyzed the expression profiles of miRNA in the serum for figuring out the differential expressed miRNAs. Serum samples from GIST patients with and without imatinib resistance and healthy controls were collected for qRT-PCR analyzing and the results showed that the content of miRNA-518e-5p in serum could distinguish imatinib-resistant GIST patients from imatinib-sensitive patients and healthy controls, which might be used as a possible biomarker for the early detection and diagnosis of GISTs and imatinib resistance by measuring miRNA-518e-5p content in serum (Kou et al., 2018). Besides, by conducting Kaplan-Meier survival analysis and log-rank analysis, expression levels of miRNA-1915 and miRNA-148b-3p were found to associate with disease-free survival (DFS) rates and overall survival (OS), and they could be regarded as biomarkers for the prognosis of GIST (Akcakaya et al., 2014; Wang Y. et al., 2018). These elementary results revealed that ncRNAs might be used as potential biomarkers to sort GIST patients in line with the prognosis for better cures.

### Therapeutic Targets

Diverse researches revealed that cell death through apoptosis pathways could be generated by the larger part of cancer chemotherapy drugs. And once the apoptosis is disorganized, drug resistance and increased cancer cell survival appear (Mohammad et al., 2015; Das et al., 2018; Mou et al., 2018). In the drug resistance of GISTs, there are a great many studies exhibiting that functions of ncRNAs on the resistance are associated with apoptosis.

LncRNA CCDC26 knocking down decreased the apoptosis of GIST cells treated with imatinib through upregulating IGF-1R, which acted in the apoptosis pathways (Li et al., 2018; Yan et al., 2019b; Zhang Y. et al., 2019). Furthermore, lncRNA CCDC26 was also reported to interact with c-KIT, thus affecting the rates of apoptotic cells caused by imatinib (Cao K. et al., 2018). BCL-2, whose gene families played pivotal roles in the programmed cell death regulations, induces apoptosis evasion and drug resistance evolution in cancers (Ashkenazi et al., 2017; Maji et al., 2018). In GISTs, BCL-2 was down-regulated by miRNA-21, miRNA-221 and miRNA-222, whose mimics were transfected in GIST cells and could significantly aggravate the apoptosis motivated by imatinib (Ihle et al., 2015; Cao et al., 2016). The overexpression of miRNA-518a-5p increased the proportion of apoptotic GIST cells treated with imatinib, which advocated that miRNA-518a-5p acted a part in interfering with the imatinib resistance (Shi Y. et al., 2016). When treated with imatinib, the resistance generated by inhibiting the apoptosis of GISTs could be attenuated by multiple ncRNAs through targeting different apoptosis-related proteins.

## Conclusion

With the researches of ncRNAs going on, an increasing number of ncRNAs in GIST have been found to play significant roles in drug resistance. This review discussed the mechanisms concerning the functions of ncRNAs in the drug resistance of GIST from five aspects: restraining the OXPHOS increased by the drugs, regulating autophagy against drug resistance, affecting the apoptosis relevant to drug resistance, changing targets of the drugs and activating relevant signaling pathways. Different types of ncRNAs on the drug resistance of GISTs were summarized. And we divided the regulating functions of ncRNAs during the drug resistance in GISTs into promotion and inhibition. Roles of ncRNAs related to different drug types and drug resistance types of GISTs were also summed up. In the end, we evaluated the potential clinical applications for ncRNAs as prognostic biomarkers and therapeutic targets. However, it remains challenging to identify the most pivotal ncRNAs from the various candidates. Identifying ncRNA that really matter in the clinical setting is of great significance for the future research, and more experiments relevant to clinic are required. Moreover, the delivery and applications of ncRNAs are difficult due to the complex internal microenvironment. Further studies on the precision and efficiency of drug delivery and translations from the experimental results into the clinical trials are urgently needed.

## Glossary

ABL: Abelson tyrosine kinase
AS: alterative splicing
asRNAs: antisense RNAs
ATG2B: autophagy-related protein 2 homolog B
Bax: BCL-2 associated X
BCL-2: B-cell lymphoma-2
BRAF: v-raf murine viral oncogene homolog B1
ceRNAs: competing endogenous RNAs
circRNAs: circular RNAs
CRISPR: clustered regularly interspaced short palindromic repeats
CSF1R: colony stimulating factor-1 receptor
DFS: disease-free survival
EMT: epithelial-mesenchymal transition
FAK: focal adhesion kinase
FGFR: fibroblast growth factor
FLT-3: Fms-like tyrosine kinase-3
GEO: Gene Expression Omnibus
GIST: gastrointestinal stromal tumor
GI tract: gastrointestinal tract
GSEA: gene set enrichment analysis
HIF-1: hypoxia-inducible factor-1
ICCs: the interstitial cells of Cajal
IGF-1R: insulin-like growth factor 1 receptor
JAK: Janus kinase
KEGG: Kyoto Encyclopedia of Genes and Genomes
KIT: KIT proto-oncogene receptor tyrosine kinase
LATS: large tumor suppressor kinase
lncRNAs: long noncoding RNAs
MEN1: multiple endocrine neoplasia type 1
miRNAs: microRNAs
MRP: multidrug resistance protein
MST: mammalian STE20-like kinase
ncRNAs: noncoding RNAs
NF1: neurofibromatosis type 1
NSCLC: non-small cell lung cancer
OS: overall survival
OXPHOS: oxidative phosphorylation
PDGFRA: platelet-derived growth factor receptor alpha
pFAK: phosphorylated FAK
piRNAs: PIWI-interacting RNAs
PI3K: phosphoinositide 3-kinase
PKB: protein kinase B
PTEN: phosphatase and tensin homolog
RAF1: raf-1 proto-oncogene serine/threonine kinase
RASSF1A: ras association domain family protein1 isoform A
Rb1: retinoblastoma susceptibility gene
RISC: RNA-induced silencing complex
rRNAs: ribosomal RNAs
SCF: stem cell factor
SDH: succinate dehydrogenase
SETD2: set domain containing 2
siRNAs: small interfering RNAs
snoRNAs: small nucleolar RNAs
STAT3: signal transducer and activator of transcription 3
TAZ: transcriptional coactivator with PDZ-binding motif
TKIs: tyrosine kinase inhibitors
tRNAs: transfer RNAs
YAP: Yes-associated protein
3′-UTR: 3′ untranslated regions
